# Molecular Dynamics to Predict Cryo-EM: Capturing Transitions and Short-Lived Conformational States of Biomolecules

**DOI:** 10.3389/fmolb.2021.641208

**Published:** 2021-04-05

**Authors:** Łukasz Nierzwicki, Giulia Palermo

**Affiliations:** ^1^ Department of Bioengineering, University of California, Riverside, CA, United States; ^2^ Department of Chemistry, University of California, Riverside, CA, United States

**Keywords:** molecular dynamics, enhanced sampling, cryo-EM, CRISPR-Cas9, structure prediction

## Abstract

Single-particle cryogenic electron microscopy (cryo-EM) has revolutionized the field of the structural biology, providing an access to the atomic resolution structures of large biomolecular complexes in their near-native environment. Today’s cryo-EM maps can frequently reach the atomic-level resolution, while often containing a range of resolutions, with conformationally variable regions obtained at 6 Å or worse. Low resolution density maps obtained for protein flexible domains, as well as the ensemble of coexisting conformational states arising from cryo-EM, poses new challenges and opportunities for Molecular Dynamics (MD) simulations. With the ability to describe the biomolecular dynamics at the atomic level, MD can extend the capabilities of cryo-EM, capturing the conformational variability and predicting biologically relevant short-lived conformational states. Here, we report about the state-of-the-art MD procedures that are currently used to refine, reconstruct and interpret cryo-EM maps. We show the capability of MD to predict short-lived conformational states, finding remarkable confirmation by cryo-EM structures subsequently solved. This has been the case of the CRISPR-Cas9 genome editing machinery, whose catalytically active structure has been predicted through both long-time scale MD and enhanced sampling techniques 2 years earlier than cryo-EM. In summary, this contribution remarks the ability of MD to complement cryo-EM, describing conformational landscapes and relating structural transitions to function, ultimately discerning relevant short-lived conformational states and providing mechanistic knowledge of biological function.

## State-of-The-Art Cryo-EM Modelling Through Molecular Dynamics

Single-particle cryogenic electron microscopy (cryo-EM) has revolutionized the field of structural biology, providing an access to the atomic resolution structures of large biomolecular complexes in their near-native environment (Nogales, 2015). The number of macromolecular structures determined by cryo-EM is rapidly increasing, indeed, it is predicted that by 2024 the number of yearly released structures will be higher for cryo-EM than for X-ray crystallography (Callaway, 2020). The cryo-EM technique comprises of three consecutive steps. At first, the sample is frozen over millisecond time scales, what results in both the formation of amorphous ice and in capturing the biomacromolecule in its near-native conformation through quick undercooling of the sample. The term “near-native” refers to the fact that during cryofixation, limited conformational transitions can result in some non-native conformations within the structural ensemble. Given the timescale of cryofixation (i.e., milliseconds), these transitions should be limited. Next, a number of two-dimensional (2D) electron microscopy (EM) images of the biomacromolecule are collected and, finally, these 2D images are combined into a three-dimensional electrostatic potential map of the biomacromolecule(Guo and Jiang, 2014; Kontziampasis et al., 2019; Cianfrocco and Kellogg, 2020). Today’s cryo-EM maps can frequently reach the atomic-level resolution, while often containing a range of resolutions, with conformationally variable regions obtained at 6 Å or worse. The latter can also arise from several other factors, such as radiation damage and image alignment errors. Moreover, considering also that the atomic form factors of cryo-EM maps represent the atomic electrostatic potential, negatively charged moieties might be depleted or not visible, as they scatter electrons more efficiently (Marques et al., 2019). Recent advances in post-processing cryo-EM images also allowed to identify multiple conformational states of the biological complexes (Jin et al., 2019) or even to describe the conformational variability of their single subunits (Bai et al., 2015). These advancements and opportunities introduced by single-particle cryo-EM are paving the way for an explosion of computational methods aimed at processing, refining and interpreting cryo-EM data (Dodd et al., 2020; Fraser et al., 2020; Kim et al., 2020; Palermo et al., 2020).

**GRAPHICAL ABSTRACT:**
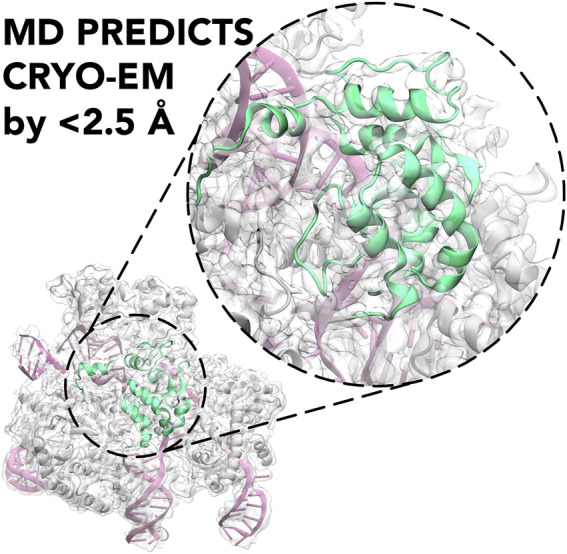
Molecular Dynamics (MD) is shown to predict the cryo-EM structure of the active CRISPR-Cas9 system with an RMSD between the cryo-EM structure and the MD ensemble of <2.5 Å.

Molecular dynamics (MD) simulations are known to be powerful in describing in detail the intrinsic dynamics of biomolecules and the energetics that underlie conformational transitions (Karplus and McCammon, 2002). This is why MD simulations are an excellent tool to examine hypotheses posed by the experimental findings of cryo-EM studies. It is also apparent that both techniques can mutually benefit from cooperation, where MD can unveil the atomic details of conformational changes and refine the structure for low resolution regions of cryo-EM maps (Kirmizialtin et al., 2015), while cryo-EM can not only provide the structure of biomolecules (Nogales, 2015), but also describe its near-native conformational ensemble in solution (Jin et al., 2019).

The initial approaches combining MD and cryo-EM methods used MD as a fitting scheme to predict the structure of a biomolecule, using the low-resolution EM map to constrain the protein conformation. For this purposes, two commonly used packages are the MD Flexible Fitting (MDFF) (Trabuco et al., 2008) and the Situs (Kovacs et al., 2018) codes, where the first one guides MD simulation toward the cryo-EM density biasing the MD potential energy form to reduce the gradient of the experimental electronic density, while the second one minimizes the discrepancy between the map derived from the MD model and the original cryo-EM map. Hybrid approaches harnessing docking algorithms have also been developed, such as including a rigid fitting stage followed by a refinement based on MD (Topf et al., 2008), or introducing a coarse-grained force field to allow flexibility during the docking search (de Vries and Zacharias ATTRACT-, 2012). MD-based methods were shown to successfully refine the structure of both isolated proteins (e.g., lactoferrin) and large protein assemblies (up to ribosomes) (Trabuco et al., 2008). Unfortunately, one of the prominent challenges for these methods is structure overfitting to the cryo-EM map, where the derived potential can lead to unphysical conformations of the biomolecule (Trabuco et al., 2009). However, such inconveniences can be overcame by combining a series of restraints derived from the experimental density with enhanced sampling MD techniques, as shown for membrane transporter Escherichia coli efflux-multidrug resistance E (EmrE) (Ovchinnikov et al., 2018). In that study, map-restrained Self-guided Langevin dynamics (Wu et al., 2013) was used with a series of heating and cooling cycles of the EmrE protein during MD run. Such approach allowed to relax both the conformation of the protein backbone and side chains and eventually led to a substantial improvement of the MD structure with respect to cryo-EM map. Enhanced sampling simulations in the structure refinement are also used in more advanced MDFF schemes, namely Cascade MDFF and Resolution Exchange MDFF (Singharoy et al., 2016). The former approach is based on simulated annealing (Brünger, 1988), where the structure is fitted sequentially to maps with higher resolution. In the latter, the Hamiltonian replica-exchange simulations (Sugita et al., 2000) are used, where in each replica the potential affecting the system is derived from the flexible fitting to the projections of the cryo-EM maps that change from low to high resolution. In this way the system is allowed to relax conformationally in low resolution replicas, while the conformations that are both relaxed in the force field and fit well to the cryo-EM maps are preferred to exchange into the high-resolution replicas. Multiple replicas were also used in a metainference method, where the restraining force arising from the difference between MD structures and the cryo-EM map is generated in an ensemble-averaged manner(Bonomi et al., 2018; Eshun-Wilson et al., 2019). Such an approach has already been shown to be fruitful in the case of NMR restraints, where the average chemical shifts or coupling constants were not necessarily representative of an heterogenous conformational ensemble present in solution (Camilloni et al., 2012). In the context of cryo-EM, this allows exploring the relevant heterogenous regions of the free energy landscape, while still remaining in agreement with the cryo-EM findings. The most recent approach, implemented in Gromacs 2020 (Igaev et al., 2019), uses a gradient of similarity between a density obtained from MD structure and the experimental density to compute the forces. This approach allows to use a variety of similarity measurements (inner product, relative entropy or cross-correlation the of the densities), enabling to adjust the density-based restraining method. Hence, one can restrain the system without enforcing the trajectory (which could lead to unphysical conformations), which helps reducing the impact of experimental artifacts (Marques et al., 2019) on the conformational dynamics of the simulated biomolecule. The method has been successfully used to unveil the origins of the SARS-CoV-2 spike protein flexibility, allowing to identify the three flexible hinges within the protein (Turoňová et al., 2020). Overall, these examples show how MD simulations guided by cryo-EM data allow for both the structure refinement the interpretation the experimental maps.

Post-processing of MD trajectories to compare the obtained structures with original cryo-EM maps can also be obtained through a variety of visualisation tools, such as e.g., Chimera (Pettersen et al., 2004) that allows for the fitting of experimental and MD derived density maps, also providing a measure for the fitting quality between densities. The recently released GROmaps tool (Briones et al., 2019) allows to compute the time-averaged MD density map and does expand a set of tools to compare the computed map with the original cryo-EM results. This method in principle can be combined with augmented Markov models (Olsson et al., 2017), where the cryo-EM map could be used as an experimental observable to reweight the simulation ensembles. Such approach increases the credibility of the comparison between cryo-EM maps and MD outcomes without biasing the simulation runs.

## Capturing Transitions and Short-Lived Conformational States

**FIGURE 1:**
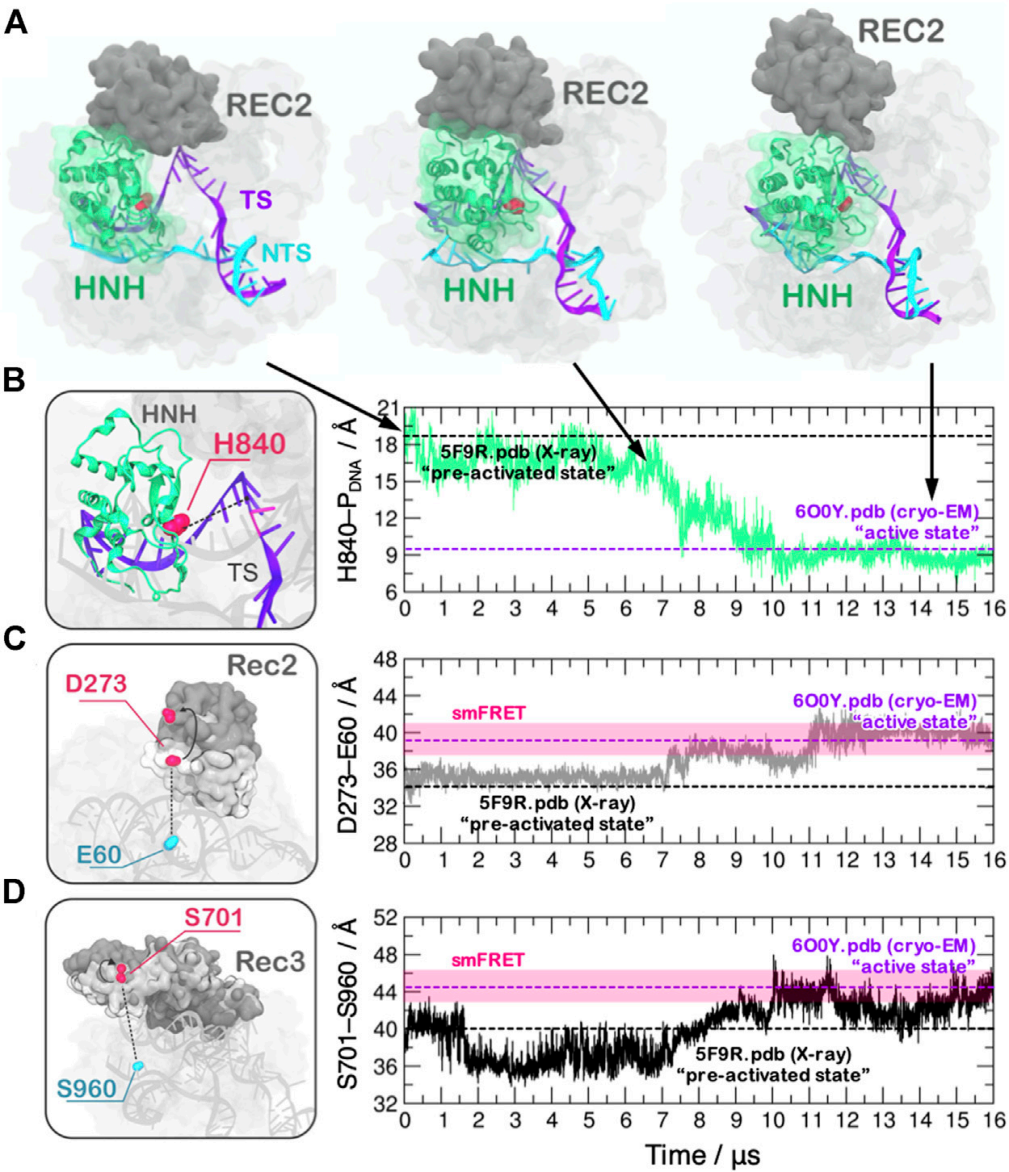
**(A)** Conformational activation of the HNH domain and structural adaptation of the REC domain during ∼16 μs of continuous MD simulations performed on the Anton-2 supercomputer (Palermo et al., 2018). **(B–D)** Time evolution of the distances: **(B)** between H840 and the cleavage site, indicating the docking of HNH at the DNA target strand; **(C)** between E60 and D273 and **(D)** between S960 and S701, indicating the opening of the REC2 and REC3 domains. Horizontal bars are used to indicate the value of the three distances in the X-ray structure of the pre-activated state (PDBid: 5F9R at 3.40 Å resolution (Jiang et al., 2016), starting configuration for MD) and in the structure obtained via cryo-EM (PDBid: 6O0Y at 3.37 Å resolution) (Zhu et al., 2019). Transparent bars indicate the distance range assumed obtained through single molecule Förster Resonance Energy Transfer experiments. Reprinted with permission from Palermo et al. (2018). Copyright 2018 Cambridge University Press. https://doi.org/10.1017/S0033583518000070.

MD can also aid cryo-EM experiments by predicting the structure of short-lived conformational states that are both essential for the biomolecular complexes activity and are hard to capture with cryo-EM because of their transient nature. A prominent example is the prediction of the active conformation of the CRISPR-Cas9 (clustered regularly interspaced short palindromic repeat and associated Cas9 proteins) system, which recently emerged as a forefront tool for genome editing (Doudna and Charpentier, 2014). At the molecular level, CRISPR-Cas9 is a large ribonucleoprotein complex, which uses RNA-guided Cas9 endonuclease to recognize and cleave matching sequences of DNA. Biophysical studies have indicated that the catalytic HNH domain is characterized by a “striking plasticity,” (Jiang et al., 2016; Palermo et al., 2016), which governs the enzymatic function. This high flexibility, however, initially hampered a definitive characterization of the catalytically competent state through cryo-EM and X-ray crystallography. Early attempts to define the structure of the catalytically active CRISPR-Cas9 employed extensive MD simulations (Palermo et al., 2017; Zuo and Liu, 2017; Palermo et al., 2018). The first effort to determine the structural transitions leading to the active state have been performed using the Gaussian accelerated MD (GaMD) method (Wang et al., 2021) that enables unconstrained enhanced sampling capturing displacements over micro- (μs) to millisecond (ms) timescales, which is of difficult reach through conventional MD. This approach described the activated state (Palermo et al., 2017). Building on this initial study, the Anton-2 supercomputer has been employed to perform unbiased runs of the complex and to determine the continuous dynamics of HNH over multiple μs (Palermo et al., 2018). This characterized the dynamical docking of HNH at the cleavage site, predicting an active conformation that confirmed the initial model obtained through GaMD (Figure 1).

This theoretical structure enabled to initiate in-depth studies of the catalysis (Palermo, 2019; Casalino et al., 2020), the allostery (Palermo et al., 2017; East et al., 2020; Nierzwicki et al., 2020) and the system’s specificity (Mitchell et al., 2020; Ricci et al., 2019), when no structural information on the active state was available. This helped obtaining information to improve the enzyme catalytic efficiency and to reduce off-target effects, which is a key goal for biomedical applications (Fu et al., 2013). The experimental determination of the catalytically competent state through cryo-EM occurred 2 years after the theoretical model (Zhu et al., 2019), reporting a remarkable agreement with the predicted model (the average RMSD between the cryo-EM structure and the MD ensemble of 2.47 ± 0.14 Å, computed considering the HNH domain and the six nucleotides at the cleavage site). Molecular simulations using Anton-2 further indicated that the recognition regions (REC) of the Cas9 protein would undergo a remarkable opening to allow the process of HNH activation (Figure 1), noting also concerted dynamics of the REC-HNH domains (Palermo et al., 2018). These coordinated domain motions were also observed through cryo-EM, revealing their functional role for DNA cleavage (Zhu et al., 2019). Furthermore, a recent single-molecule study probing the conformational dynamics of Cas9 in the post-catalytic state highlighted rapid conformational fluctuations of HNH (Wang et al., 2021), as observed through MD. These results highlight the consistency of the simulations with experimental observations and suggest that state-of-the-art MD can capture short-lived conformational states of biomolecules, which are of difficult reach through structural biophysics techniques.

## Summary and Perspectives

Here, we highlighted how MD simulations combined with cryo-EM data can provide a deep understanding of key conformational steps that govern the function of biomacromolecules. MD can be used not only to refine cryo-EM structures, especially the low-resolution regions, but also to facilitate interpretation of the experimental findings. Novel MD analysis tools allow also to compute the time-averaged cryo-EM maps from MD trajectories, enabling a reasonable comparison between conformational ensembles determined experimentally and computationally. This overcomes the limitations of comparing single structures, lacking of dynamical information. Finally, MD simulations alone were also shown to be a powerful predicting tool, that allows to characterize the short-lived conformational states of biomolecules hard to capture through cryo-EM.

Ultimately, the rapid development of methods that combine cryo-EM data with MD will further increase the reliability of MD-guided predictions. One can expect that the rigorous comparison between cryo-EM and MD conformational ensembles can be an additional source of the data that can be used to improve the currently available simulation methods. Molecular simulations can also be guided to a conformational ensemble defined as a cryo-EM map rather than a specific structure. This can improve the description of the free energy landscape associated with conformational changes of proteins and nucleic acids, as the cryo-EM map can be used as a reference for the conformational ensemble. Such approach, based on Multi-Map variable method, was very recently released for NAMD (Vant et al., 2020). The initial results for both the steered-MD simulations and free energy methods are encouraging, with the free energy profiles for the conformational transitions comparable to those determined using high-resolution structures as a reference. Overall, non-stop development of cryo-EM–based MD methods opens novel opportunities for the precise description of biomolecular dynamics.
