# Corrigendum: Molecular Dynamics to Predict Cryo-EM: Capturing Transitions and Short-Lived Conformational States of Biomolecules

**DOI:** 10.3389/fmolb.2021.698735

**Published:** 2021-05-28

**Authors:** Łukasz Nierzwicki, Giulia Palermo

**Affiliations:** ^1^ Department of Bioengineering, University of California, Riverside, CA, United States; ^2^ Department of Chemistry, University of California, Riverside, CA, United States

**Keywords:** molecular dynamics, enhanced sampling, cryo-EM, CRISPR-Cas9, structure prediction

In the published article, there is an error in the Funding statement. The correct number for the National Institute of Health is R01GM141329. The authors apologize for this error and state that this does not change the scientific conclusions of the article in any way. The original article has been updated.

